# Decision uncertainty and value of further research: a case-study in fenestrated endovascular aneurysm repair for complex abdominal aortic aneurysms

**DOI:** 10.1186/s12962-018-0098-7

**Published:** 2018-04-16

**Authors:** Oriana Ciani, David Epstein, Claire Rothery, Rod S. Taylor, Mark Sculpher

**Affiliations:** 1 Evidence Synthesis and Modeling for Health Improvement, Institute of Health Research, University of Exeter Medical School, South Cloisters, St Luke’s Campus, Exeter, EX1 2LU UK; 2 Center for Research on Health and Social Care Management, SDA Bocconi University, via Roentgen 1, 20136 Milan, Italy; 3 Centre for Health Economics, University of York, Heslington, Alcuin ‘A’ Block, York, YO10 5DD UK; 4 Department of Applied Economics, University of Granada, Campus Universitario de Cartuja, 18071 Granada, Spain

**Keywords:** Cost-effectiveness, Coverage with evidence development, Medical devices, Value of information

## Abstract

**Background:**

Fenestrated endovascular aneurysm repair (fEVAR) is a new approach for complex abdominal aortic aneurysms, limited to a few specialist centers, with limited evidence base. We developed a cost-effectiveness decision model of fEVAR compared to open surgical repair (OSR) to investigate the likely direction of costs and benefits and inform further research projects on this technology.

**Methods:**

A systematic review with meta-analysis and a four-state Markov model were used to estimate the cost-effectiveness of fEVAR versus OSR. We used a recent coverage with evidence development framework to characterize the main sources of uncertainty and inform decisions about the type of further research that would be most worthwhile and feasible.

**Results:**

Seven observational comparative studies were identified, of which four presented odds ratios adjusted for confounders. The odds ratios for operative mortality varied widely between studies. Assuming a central estimate of the odds ratio of 0.54 (95% CI 0.05–6.24), the decision model estimated that the incremental cost per quality adjusted life year (QALY) was £74,580/QALY with a probability of 9 and 16% of being cost-effective at standard cost-effectiveness thresholds of £20,000/QALY and £30,000/QALY, respectively. The Expected Value of Perfect Information over 10 years at a threshold of £20,000/QALY was £11.2 million. Operative mortality contributed to most of the uncertainty in the decision model.

**Conclusions:**

In the case of “maturing technologies”, decision modelling indicates the likely direction of costs and benefits and guides the development of further research projects. In our analysis of fEVAR versus OSR, decision uncertainty, particularly around operative mortality, might be effectively resolved by a short-term RCT, or possibly a well-conducted comparative observational study. Decision makers may consider that a conditional coverage decision is warranted with assessments required to make this type of recommendation depending on local priorities and circumstances.

**Electronic supplementary material:**

The online version of this article (10.1186/s12962-018-0098-7) contains supplementary material, which is available to authorized users.

## Background

Health technology assessment (HTA) agencies are charged with providing guidance to practitioners and patients based on a systematic and comprehensive assessment of the clinical and cost-effectiveness profile of health technologies. Assessing medical devices (MDs), in particular, raises several challenges which require careful consideration [1]. For a range of reasons, evidence on costs and effects relating to medical devices may be limited [2]. This situation, usually but not exclusively associated to MD evaluation, leads to a dilemma that typically has proved challenging for HTA and policy makers. Making an early decision based on poor evidence carries a high risk of error that may be difficult to reverse, but delaying a decision until more evidence becomes available may leave current patients without effective treatment or health systems with inefficient technology.

Fenestrated endovascular repair fEVAR is an example of such dilemma. Fenestrated EVAR might be considered a maturing innovation in the typology of Sculpher et al. [3], that is a technology early in its life cycle, usually limited to a few specialist centers, with an evidence base comprising case series and small RCTs. Decision modelling in these situations is unlikely to provide a definitive statement about whether a technology is cost-effective, but rather indicate the likely direction of costs and benefits, the circumstances under which the new technology might represent good value for money, and to guide the development of further research projects [3]. The aim of the study is to illustrate this approach through the clinical and cost-effectiveness evaluation of fEVAR compared to open surgical repair and to estimate  the value of further research for the same decision problem.

Abdominal aortic aneurysms (AAAs) occur when the main artery in the body develops a balloon-like bulge of diameter exceeding 3 cm. The prevalence of AAAs in the UK has been estimated at between 1.2 and 7.6% in those over 50 years [4]. The benefits of endovascular aneurysm repair (EVAR) compared to open surgical repair (OSR) of AAAs are well established by large multicenter randomized trials assessing all-cause or aneurysm related mortality and postoperative complications [5–7]. However, about 15% of AAAs are considered “complex” (cAAAs), i.e. aneurysms involving renal or visceral arteries or ‘juxta-renal AAAs’ [8]. Surgical treatment options for cAAAs include OSR, usually requiring suprarenal aortic cross-clamping, or fEVAR [8–10]. In fEVAR the stent-graft fabric extends over the renal arteries, but perfusion to these arteries is preserved via accurately placed windows (fenestrations) within the stent-graft fabric. The fEVAR procedure is more challenging than standard EVAR as graft positioning requires both longitudinal and rotational alignment of the fenestrations with the target vessels [11].

Although a small number of systematic reviews have sought to compare efficacy and safety outcomes of fEVAR compared to OSR, their conclusions have been limited by lack of available robust evidence [12–14]. In particular, these reviews noted that there were no randomised controlled trials (RCTs), but there were several registries and case series, some of which were large and long-running.

This study evaluates the effectiveness, safety and cost-effectiveness of fEVAR versus OSR for elective repair of cAAA in patients suitable for either procedure. Another solution for more complicated aneurysms is branched EVAR (bEVAR). The term ‘branched’ refers to the need to bridge the gap, created by increased diameter of aorta, between the main body of aortic stent graft and target vessels and not to any actual branch from the graft itself [11]. Based on the distinction between bEVAR and fEVAR in accordance with the Society of Vascular Surgery reporting standards on thoracic endovascular aortic repair [10], branched cAAAs endovascular procedures were excluded from this analysis.

## Methods

### Systematic review and meta-analysis

This updated systematic review was conducted and reported according to the Meta-analysis of Observational Studies in Epidemiology (MOOSE) reporting guidelines [17].

#### Data sources and search strategy

MEDLINE (via Ovid) was searched for previous systematic reviews or meta-analyses of fEVAR for the treatment of cAAAs. We also sought new primary studies published since the end date of the literature searches of the most recently undertaken systematic review (i.e. October 2013) [18] and up to 14th January 2015. A copy of the search strategy is available as an Additional file 1: Appendix S1. As this recent review found no RCTs, this updated study also includes observational evidence. Two reviewers (OC, RST) independently examined titles and abstracts of primary studies.

#### Study selection

Relevant articles were obtained in full, and assessed against the inclusion/exclusion criteria described in Additional file 1: Table S1. Any disagreement was resolved through discussion.

#### Data extraction and risk of bias assessment

Details were extracted for each study including: year and country of publication, sample size, age and gender distribution, duration of follow up, anatomical location of aneurysms, and details of implant used. Outcomes of interest were: operative mortality, late mortality, complications, re-interventions, and resource use during the primary admission (i.e. length of stay, operative time, blood, and intensive care). Study quality was assessed using a modified list of criteria developed for case series [19] and the addition of three criteria for comparative studies. Data extraction and risk of bias assessment was undertaken by one author (OC) and verified by another (RST).

#### Data analysis

Where outcome data were consistently reported, results were pooled across studies using Der Simonian and Laird random-effects meta-analyses [20]. For operative mortality, we considered that treatment effects should only be pooled where authors made statistical adjustment for relevant confounding factors. For resource use, we considered that unadjusted differences were adequate as these variables are relatively less influenced by factors such as body mass index, leukocyte count or other biomarkers. Moreover, no studies reported between-groups adjusted comparison of these measures. Recommendations from the Cochrane Handbook for systematic reviews of interventions were followed to deal with missing data [20]. Heterogeneity was quantified using the I^2^ statistic and results displayed in a forest plot. All statistical analyses were performed using STATA^®^ 13.1 StataCorp US.

### Cost-effectiveness model

This economic evaluation was undertaken and reported according to the Consolidated Health Economic Evaluation Reporting Standards (CHEERS) statement [21].

#### Model structure

**Fig. 1:**
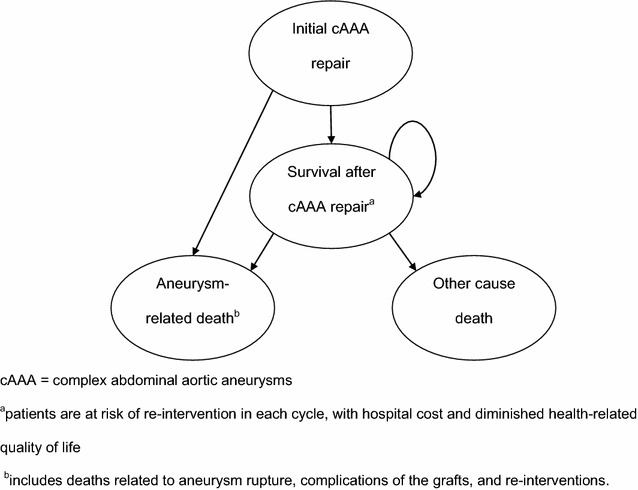
Decision analytic model structure. *cAAA* complex abdominal aortic aneurysms. Patients are at risk of re-intervention in each cycle, with hospital cost and diminished health-related quality of life. Includes deaths related to aneurysm rupture, complications of the grafts, and re-interventions

A decision analytic model was developed in Excel^®^2012 (Microsoft Corporation, US) to estimate the lifetime cost and QALY of each procedure. The structure of the model was adapted from a previously published economic evaluation of conventional EVAR for AAA repair, using essentially the same structure, with parameters estimated from the systematic review [22]. The model consists of a lead-in period lasting 6 months, during which the cAAA repair is undertaken with fEVAR or OSR (Fig. 1). Perioperative survivors then enter a long-term three state Markov model, which extrapolates costs and outcomes over a lifetime horizon using 6-monthly cycles. During each cycle, patients may die of cAAA-related causes, such as rupture of the aneurysm, or die of other causes. Patients undergo surveillance with an outpatient visit and ultrasound every year after endovascular repair and every 5 years after open repair [23]. Survivors have an ongoing risk of re-intervention, e.g. to correct complications of the device such as endoleaks which might be detected by surveillance. Patients can have only one re-intervention per cycle, but can have more than one re-intervention over their lifetime. The risk of re-intervention depends on initial procedure (OSR or fEVAR) and time from initial cAAA repair, but is assumed independent between cycles. Re-interventions reduce health-related quality of life (HRQOL) and incur a hospital cost. Mortality risks related to re-intervention are captured in the estimate of aneurysm-related deaths.

#### Perspective of the decision model and inputs required

Health outcomes were expressed in terms of quality-adjusted life years (QALYs). The perspective adopted was that of a publicly funded health system using an explicit cost-effectiveness threshold for decision making. The UK National Health Service (NHS) [24] is used here as an example, using thresholds in the range £20,000-£30,000 per QALY. The price year was 2016/17, and costs and benefits were discounted at an annual rate of 3.5% [24].

Table 1 summarises the main input variables required by the decision model and their distributions used to represent uncertainty. Parameter estimates for the model were obtained from the updated systematic literature review, personal communication with the device manufacturer, and routine UK National Health Service (NHS) sources. Where specific estimates for fEVAR were unavailable, the literature on conventional EVAR was considered. The mean age at which cAAAs repair is undertaken was 73 years [25]. Mortality risks for non-AAA cause deaths were based on UK male population life tables [26].

**Table 1:** Mortality, re-intervention and HRQoL in the cost-effectiveness decision model

	Mean value used in the model	Distribution used in the model	Source
Early mortality			
Probability of AAA-related mortality, fEVAR, 0–6 m	Pr = 0.026	Beta (43,1627)	Additional file 1
Log-odds ratio, fEVAR vs OSR	− 0.61	N (Mean − 0.61, SE 1.22)	Meta-analysis of 4 studies (adjusted for case-mix)
Late AAA mortality			
Late mortality rate > 6 months–4 years, fEVAR	0.004	Gamma (6, 1/1558)	Additional file 1
Late mortality rate > 4–8 years, fEVAR	0.006	1.5 × 6 months–4 years	EVAR-1
Late mortality rate > 8 years, fEVAR	0.008	2 × 6 months-4 years	EVAR-1
Log Hazard ratio > 6 months-4 years, fEVAR vs OSR	0.38	N (0.38, 0.48)	EVAR-1
Log Hazard ratio > 4–8 years, fEVAR vs OSR	1.13	N (1.13, 0.57)	EVAR-1
Log Hazard ratio > 8 years, fEVAR vs OSR	1.76	N (1.76, 0.63)	EVAR-1
Re-interventions 0–6 months			
Probability, fEVAR	0.068	Beta (81, 1117)	Additional file 1
Log odds ratio, fEVAR vs OSR	− 0.87	N (− 0.87, 0.24)	Meta-analysis of 3 studies (unadjusted for case-mix)
Re-interventions > 6 months			
Re-intervention rate > 6 months-4 years, fEVAR	0.087	Gamma (132, 1/1507)	Additional file 1
Re-intervention rate > 4–8 years, fEVAR		As > 6 months–4 years	Assumption
Re-intervention rate > 8 years, fEVAR		As > 6 months–4 years	Assumption
Log Hazard ratio > 6 months–4 years, fEVAR vs OSR	1.84	N (1.84, 0.35)	Additional file 1
Log Hazard ratio > 4–8 years, fEVAR vs OSR	0.47	N (0.47, 0.34)	EVAR-1
Log Hazard ratio > 8 years, fEVAR vs OSR	0.47	N (0.47, 0.34)	EVAR-1
Health related quality of life, EQ-5D index			
After AAA repair, baseline	0.804	N (0.804, 0.021)	EVAR-1
Decrement after OSR 0-6 m	− 0.060	N (− 0.060, 0.017)	EVAR-1
Decrement in the 6 months following a re-intervention	− 0.060	N (− 0.060, 0.026)	EVAR-1
Decrement in the 6 months before death	− 0.149	N (− 0.149, 0.017)	EVAR-1

*AAA* Abdominal aortic aneurysms; *OS*R Open surgical repair; *m* months; *yr* years

#### Expected value of perfect information

Fenestrated EVAR is a relatively novel technology and the evidence relating to its costs and benefits is limited. In this regard, further research might be worthwhile if it reduces uncertainty and avoids the consequences of making a wrong decision about the use of the intervention. The expected value of perfect information (EVPI) estimates an upper bound for the value of a new study, if it could eliminate all uncertainty about which treatment was more cost-effective at the given cost-per-QALY threshold. Partial EVPI (EVPPI) is also calculated using web-based emulator software [27]. EVPPI enables identification of those parameters that contribute particularly highly to decision uncertainty.

A decision timeframe of 10 years is used, to allow for obsolescence and product innovation by competing technologies. We assume that 1782 cAAA patients would be eligible for repair each year in the UK, based on German health insurance data [25] adjusted for UK population size. Net population benefit was calculated at cost-effectiveness thresholds used in the UK.

Decision uncertainty was estimated through probabilistic sensitivity analysis using 1000 Monte-Carlo simulations of the model [28].

#### Decision making and the value of further research

We used a recently developed framework to characterize the sources of uncertainty and to identify the most appropriate decision option for fEVAR given the available evidence [15, 29]. Four decision options were considered: accept fEVAR for immediate and general use in the national heath service in indicated patients (accept fEVAR), reject fEVAR (i.e. accept OSR), accept fEVAR provisionally but conditioned upon the collection of further evidence (accept with research, AWR), or reject fEVAR for general use until further evidence becomes available (only in research, OIR).

## Results

### Systematic review and meta-analysis

**Fig. 2:**
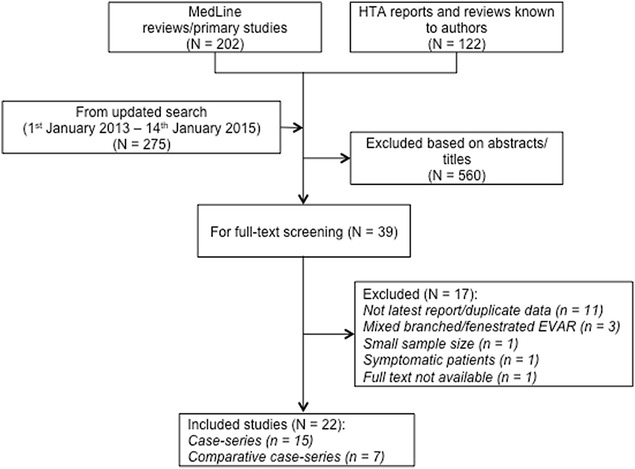
Flow diagram—study selection process

The updated systematic review study selection process is illustrated in Fig. 2. A total of 22 studies involving 1641 fEVAR patients were included (see Additional file 1: Appendix S2 for list of included studies). Whilst we did not find any randomised trials comparing fEVAR and OSR, we did include seven comparative observational studies, reporting outcomes for both fEVAR and OSR patients [30–36].

### Study characteristics

A summary of the study and patient population characteristics and the interventions is reported in Additional file 1: Table S2. Study sample sizes were small, with a median of 78 fEVAR treated patients, a median patient age of 74 years and a median follow-up of 18.4 months. All studies showed high to moderate risk of bias as reported in Additional file 1: Table S3.

### Relative risk of operative mortality

Seven observational studies compared perioperative [30, 36] or 30-day [31–35] mortality between fEVAR and OSR (Additional file 1: Table S4). The pooled odds ratio for mortality across all seven studies was 0.60 (95% CI 0.23–1.60, p = 0.014) (Additional file 1: Figure S1). However, there was a high degree of heterogeneity between studies (I^2^ statistic: 62.6%, p = 0.014).

### Risk of bias for estimate of relative risk of operative mortality

In a context of poor available evidence, it is even more important to explore the heterogeneity and evaluate the risk of bias in order to reliably use the information for decision-making purposes. One source of heterogeneity was the method used by the study to account for possible confounding. Three studies did not make any adjustment for confounding variables [31–33]. Of the four that took account of confounding variables in some way, one used multivariate regression [35], one used propensity score matching [34], and two used a published risk score [37] to estimate what operative mortality hypothetically would have been in the fEVAR patients had they had undergone open repair [30, 36]. More on the risk of bias assessment performed for estimate of relative risk of operative mortality is reported in Additional file 1: Appendix S3.

### Relative risk of operative mortality stratified by study methodology

To further explore the heterogeneity, we stratified the pooled estimate of effect size according to the methodology in the study employed to adjust for confounders. The Raux study [34] (propensity score matching) found an OR of 5.1 (95% CI 1.1–24). The Tsilimparis study [35] (multivariate regression) found an OR of 0.19 (95% CI 0.04–0.83). The two studies that used the V-POSSUM risk score 26,32 found a pooled OR of 0.37 (95% CI 0.20–0.69). The remaining studies that did not adjust for confounders found a pooled OR of 0.98 (95% CI 0.26–3.7).

### Relative risk of operative mortality used as input to the decision model

**Fig. 3:**
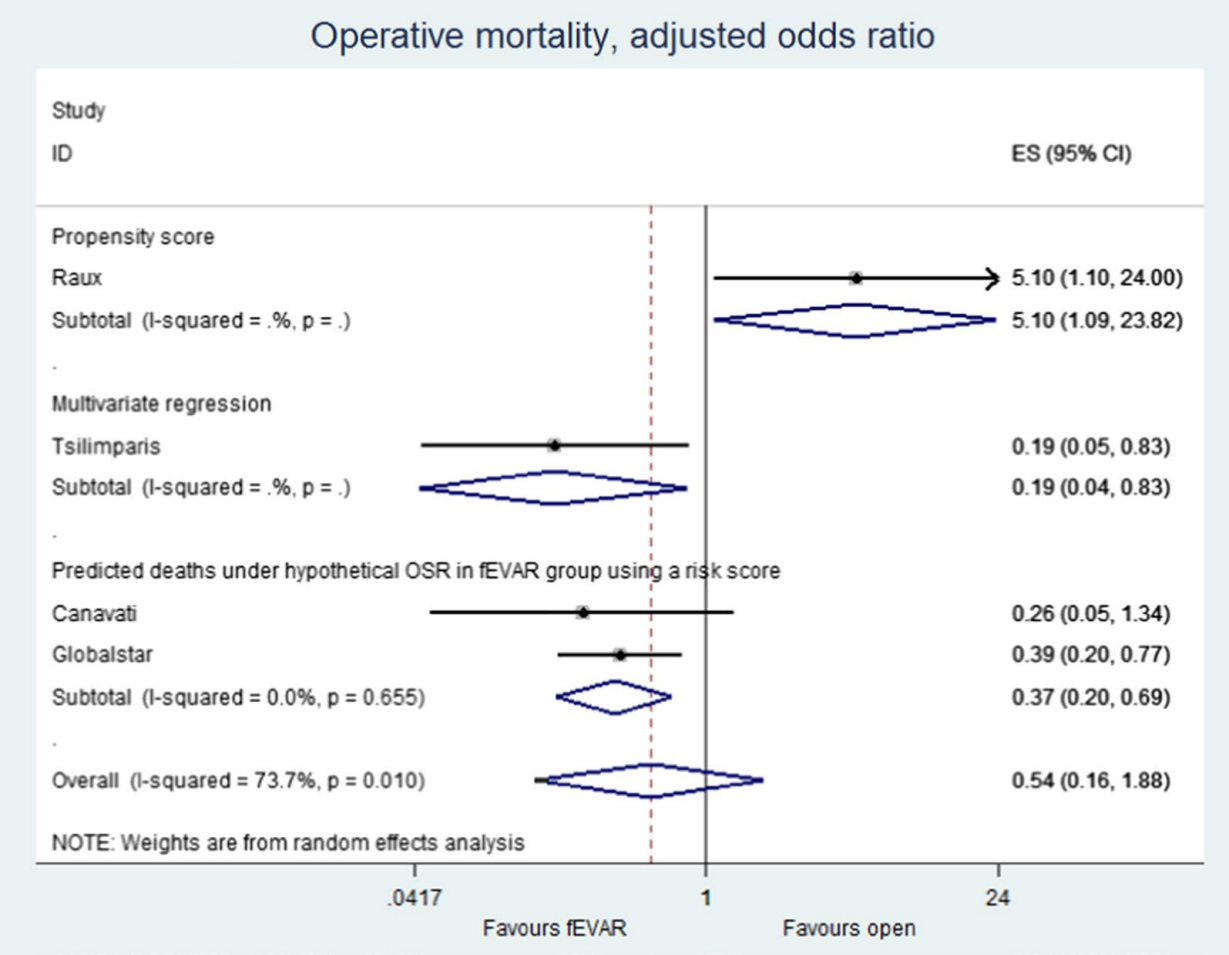
Pooled odds ratio of adjusted operative mortality fEVAR vs OSR

The pooled estimate of the odds ratio over the four studies that performed some form of risk adjustment is 0.54 (95% CI 0.16–1.88) (Fig. 3). The decision model uses the OR estimate of 0.54 as the base-case (log(OR) = μ = − 0.61, SE(μ) = 0.644). However, this SE from random-effects meta-analysis does not take account of the full distribution of effect sizes across the heterogeneous studies [38]. In order to make predictions about the likely effect size that might occur in a future new study or in the general practice use, we considered the estimate of τ^2^ from the four adjusted studies in the random-effects meta-analysis [30, 34–36] (τ^2^ = 1.15). This suggests a mean and confidence interval for a prediction for a new study of 0.54 (95% CI 0.05–6.24).

### Baseline risk of operative mortality used as input to the decision model

Twenty-two studies reported operative mortality after fEVAR, with 43 deaths in 1670 patients (mean rate 2.6%) [30–36, 39–53]. However, there was considerable heterogeneity, with operative mortality varying from 0% to 9.5% (Additional file 1: Table S5). Analysis of UK national hospital episode statistics indicates the mean operative mortality after OSR in clinical practice, as opposed to selected case series in specialist centres, is 14%, with considerable heterogeneity [54]. In the model, we use the mean operative mortality after fEVAR from the systematic review as the probability of AAA death during the first model cycle in the base-case and vary this from 0.5% to 9.5% in sensitivity analyses, assuming the baseline rate and the relative risk are independent.

### Late aneurysm-related mortality

One study included in the review compared late aneurysm related mortality with about 1 year of follow-up between fEVAR and OSR [31] and found no late AAA-related deaths in either group.

Thirteen studies [31, 32, 36, 39–42, 44–46, 49, 51, 52] reported late AAA mortality after fEVAR. The overall rate of late AAA mortality was 6 deaths in 1558 patient-years (i.e. an average of 0.004 late AAA deaths per person-year) although 10 studies [36, 39–47] reported no late AAA-related deaths (Additional file 1: Table S5). The decision model assumes this 0.004 rate of late AAA mortality after fEVAR from > 6 months to 4 years (as found by this review), but that this rate would increase by 50% between 4 years and 8 years and doubles after 8 years (as found after conventional EVAR by the EVAR-1 trial [22]). Furthermore, the base-case model assumes relative risk of late AAA mortality would be greater after fEVAR compared with OSR (as found by the EVAR-1 trial [22]). Sensitivity analyses explore a scenario where the rate of late AAA mortality does not increase over time and is the same as OSR (as found by Chisci et al. [31] study at 1 year) over the patient’s lifetime.

### Early and late re-interventions

Eleven studies reported a total of 81 early re-interventions (in-hospital or 30-day) in 1198 patients after fEVAR, an average probability of 0.07 [30, 31, 34, 39, 42, 43, 47, 50, 52]. Three studies compared the risk of early (in-hospital or 30-day) re-interventions for fEVAR compared to open repair [30, 31, 35]. All found more re-interventions after open repair with a pooled OR across the three trials of 0.42 (95% CI 0.26–0.68) (Additional file 1: Figure S4). These results were not adjusted for case-mix. Eleven studies reported a total of 132 late re-interventions in 1507 patient-years of follow-up after fEVAR, an average rate of 8.8 per 100 patient-years. No trial reported late re-interventions after open repair [32, 36, 41, 47, 48, 51, 52].

As the studies did not provide sufficient information on how the re-interventions rate might change over time, the decision model uses the rates of early re-interventions from the literature review, and the relative risk of late re-intervention (fEVAR vs OSR) from the EVAR-1 RCT [22] that compared EVAR versus open repair for conventional AAA, which was 6.29 (95% CI 3.09–12.78) from 6 months to 4 years, 1.60 (95% CI 0.81–3.15) from 4 to 8 years, and 1.51 (1.71–3.19) after 8 years [55].

### Resource consumption

Five studies provided comparative information on one or more healthcare resource use outcomes (Table 2, Table 3) [30–33, 35]. There was considerable heterogeneity across most of the resource use data. The decision model uses the average estimates of procedure resource use (i.e. length of stay, operating theatre, blood units and Intensive Care Unit stay) costed at UK prices in 2016/17 [55, 56]. The list price of the fEVAR device was £16,500 including extra stent parts and accessories (personal communication with Cook Medical, at 2015 prices). Sensitivity analyses were conducted by varying the price of the stent to reflect possible discounts. A reduction in cost might also be achieved if use of fEVAR was associated with lower length of stay or less use of other hospital resources, or if surveillance and re-interventions could be accurately targeted at patients with higher risk of complications. Other unit costs associated with the index procedure are given in Table 3. The cost of a AAA-related re-intervention was taken as £8670 (SE 831) [55].

**Table 2:** Resource consumption in fEVAR and OSR patients

	fEVAR		OSR		Pooled WMD (95% CI)
	N	Mean (SD)	N	Mean (SD)	
Length of stay (days)					
Moore 2006	16	14.8 (17.3)	29	13 (8.0)	− 4.83 (− 7.33 to − 2.32) I^2^ = 87.4% p < 0.0001
Donas 2012	29	3.5 (1.1)	31	7.2 (3.2)	
Canavati 2013^c^	53	7.0 (3.3)	54	12 (9.6)	
Tsilimparis 2013	246	3.0 (4.3)	1091	10 (9.6)	
Operative time (min)					
Moore 2006	16	268 (113.0)	29	205 (196.0)	38.72 (− 79.00 to 156.44) I^2^ = 98.6% p < 0.0001
Chisci 2009*	52	266 (94)	61	150 (38)	
Donas 2012	29	290 (122.0)	31	204.00	
Canavati 2013	53	300 (88.9)	54	235 (66.7)	
Tsilimparis 2013	246	175.7 (101.8)	1091	260.57 (109.0)	
Blood unit transfused^b^					
Moore 2006	16	4.4 (5.8)	29	6.5 (5.8)	− 2.41 (− 2.72 to − 2.11) I^2^ = 16.1% p = 0.311
Chisci 2009^ac^	52	1.12 (0.95)	61	3.45 (1.24)	
Donas 2012	29	0.2	31	3.2 (1.2)	
Canavati 2013^ac^	53	2.8 (2.2)	54	4.4 (3.1)	
Tsilimparis 2013	246	0.5 (1.9)	1091	3.1 (4.0)	
ICU stay (days)					
Moore 2006	16	4 (8.5)	29	1.7 (2.7)	− 0.88 (− 6.8 to 5.04) I^2^ = 79.2% p = 0.028
Canavati 2013^ac^	53	0.58 (7.8)	54	4.33 (26.7)	

*ICU* intensive care unit; *WMD* weighted mean difference

^a^Median instead of mean

^b^450 ml blood per unit

^c^IQR = 1.35*SD as in normal distribution

**Table 3:** Costs of procedures in the economic model

	Natural units, OSR	Difference fEVAR-OSR	Unit cost (£)	Mean cost open	Mean cost fEVAR
Resource	Mean of studies from meta-analysis	Pooled WMD from meta-analysis (Table 2)	53		
Length of stay (days)	10	− 4.83	353	2593	1352
Operative time (mins)	251	38.72	19.58	4421	5102
Blood units (450 ml)	3.25	− 2.41	144	454	118
ICU stay (days)	3.41	− 0.88	1178	4059	3012
Cost of device (£)				312	16,502
Other consumables				246	516
Total				13,529	27,658

Costs inflated to 2016/17 prices using hospital pay and prices index [56]

*WMD* weighted mean difference, *OSR* open surgical repair, *fEVAR* fenestrated endovascular repair

### Health-related quality of life

No study reported health related quality of life (HRQOL) after fEVAR or OSR. The EVAR-1 trial found that HRQOL was better after conventional EVAR than OSR for the first 6 months (mean difference in EQ-5D-3L 0.060, SE(0.017)) but there was no difference by 1 year [22]. HRQOL is considerably diminished in the 6 months after a re-intervention and in the 6 months preceding death [57].

### Complications

Peri-operative complications were poorly and heterogeneously reported, especially after open repair. The studies did not give a clear indication of the seriousness of the complication or its duration. Therefore complications were not explicitly costed or associated with QALYs in the model. However, some measure of their impact is included in the cost-effectiveness analysis, because serious complications and adverse events during the surgical procedure and hospital stay will be reflected in HRQOL, length of stay, operative theatre time, blood use, and use of ICU.

### Cost-effectiveness model

The mean difference in lifetime cost between fEVAR and OSR was £15,606 (95% CI 8390–22,512), and the lifetime difference in QALY was 0.209 (95% CI − 0.124–1.311), (Table 4, Additional file 1: Figure S2). The incremental cost-per-QALY is estimated to be £74,579/QALY with a probability of 0.090 of being cost-effective at a threshold of £20,000 per QALY, and a probability of 0.158 at £30,000 per QALY [24]. Almost all of the additional cost is incurred in the price of the fEVAR device (Additional file 1: Figure S3).

**Table 4:** Mean costs and QALYs estimated by the model and sensitivity analyses

	Cost OSR	QALY OSR	Cost fEVAR	QALY fEVAR	Difference in cost	95% CI		Difference in QALY	95% CI		ICER	p (20 k)	p (30 k)
Base-case	16,067	4.333	31,673	4.542	15,606	8391	22,512	0.209	− 0.124	1.311	74,580	0.090	0.158
Model2	16,246	4.626	31,604	4.544	15,358	8126	22,902	− 0.082	− 0.167	0.090	Dominated	0.001	0.003
Model3	15,860	4.017	31,694	4.554	15,834	8383	23,611	0.537	− 0.072	1.944	29,498	0.269	0.401
Model4	15,801	3.923	31,648	4.295	15,848	8467	23,859	0.372	− 0.318	1.707	42,584	0.224	0.318
Model5	16,194	4.525	31,762	4.615	15,567	8126	23,379	0.090	− 0.083	0.612	173,452	0.019	0.046
Model6	16,032	4.303	31,876	4.547	15,843	8438	23,193	0.243	− 0.071	1.136	65,154	0.077	0.165
Model7	18,292	4.353	31,665	4.548	13,373	6089	20,803	0.196	− 0.118	1.101	68,348	0.100	0.169
Model8	16,047	4.344	22,948	4.545	6901	− 804	14,238	0.201	− 0.123	1.308	34,373	0.249	0.330

*OSR* open surgical repair, *fEVAR* fenestrated endovascular aneurysm repair, *ICER* incremental cost effectiveness ratio, *QALY* quality-adjusted life years, *Pr(X)* probability that fEVAR is cost-effective at threshold of X(£), *Dominated* fEVAR had greater cost and lower health benefit

Scenario 2: Odds ratio (OR) of operative mortality favours OSR

Scenario 3: OR of operative mortality is more favourable to fEVAR than the base-case

Scenario 4: Greater base-line risk of operative mortality

Scenario 5: Lower base-line risk of operative mortality

Scenario 6: No difference in late-aneurysm related deaths between groups

Scenario 7: No difference in late re-interventions between groups

Scenario 8: Lower price of endovascular device

#### External validation of model results

The predicted overall survival in the model after OSR at 5 years is 58% (Additional file 1: Figure S2), the same as reported from a study linking Hospital Episode Statistics to national mortality records in the UK [54].

#### Sensitivity analyses

The following univariate sensitivity analyses were conducted:

1. Base-case;
2. High relative risk of operative mortality (OR 5.1, 95% CI 1.10–24) [34];
3. Low relative risk of operative mortality (OR 0.19, 95% CI 0.05–0.83) [35];
4. High baseline risk of operative mortality after fEVAR of 9.5% [34];
5. Low baseline risk of operative mortality after fEVAR of 1% [35];
6. No difference in late (> 30 day) mortality between fEVAR and open repair [31];
7. No difference in late re-interventions between fEVAR and open repair;
8. Price of fEVAR device of £7500.

The sensitivity analyses show the circumstances under which fEVAR plausibly might be cost-effective (Table 4). If the OR of operative mortality were that observed in the most favourable study, assuming that baseline risk remains unchanged, then fEVAR comes close to the £30,000/QALY threshold. fEVAR would also be close to this threshold if the cost of the device were £7500, rather than £16,500.

#### Expected value of perfect information

The EVPI over 10 years at a threshold of £20,000 per QALY was £11.2 million. EVPPI analysis showed that 92% of this value could be attributed to the two operative mortality parameters (baseline and odds ratio), and the remainder to the cost of the procedure.

#### The value of further research

Under the base-case model, fEVAR is unlikely to be cost-effective. This does not necessarily mean that it should be rejected, if further evidence might lead to different conclusions. Claxton et al. provide a checklist of the sequence of assessments that would be required for an HTA body considering whether further research should be mandated [58]. We provide a structured discussion of the issues as they might apply to fEVAR given the results of this study. However, each decision maker would need to review and interpret the data as they apply to health services in their jurisdiction.

#### Is the technology expected to be cost-effective?

This would depend on the decision criteria used (e.g. the cost-per-QALY threshold) as well as the decision-maker’s interpretation of the appropriate inputs for the model. We assume it is likely not cost-effective on current evidence and from the perspective of the NHS in the UK [24].

#### Are there significant irrecoverable costs?

Implementing FEVAR would not require significant irrecoverable costs in NHS infrastructure, if most of the up-front investment in operating facilities and interventional radiography has already been made. However, on-going training for surgical teams would be required, and the procedure is of course irreversible for individual patients.

#### Does more research seem worthwhile?

There are no RCTs, there is considerable decision uncertainty from the model, and more research might plausibly reduce that uncertainty. Some assessment is therefore needed of the type of evidence and whether it can be conducted without approval.

#### Is the research possible with (without) approval?

Generally, most types of research are possible without approval, including RCTs. However, the patient population who might potentially benefit from fEVAR is small and hence population EVPI is only about £11 million at a threshold of £20,000/QALY in the UK. This means a large-scale, expensive, lengthy RCT would not be cost-effective. Nevertheless, in our EVPPI analysis, most of the uncertainty was around operative mortality, which might indicate that a RCT with short follow-up, or even a well-designed observational study would provide sufficient evidence for definitive approval or rejection. These factors would indicate OIR would be an appropriate decision.

#### Will other sources of uncertainty resolve over time?

A further source of uncertainty, which has not been reflected in our analysis, is the possibility of a learning curve for fEVAR. Fully understanding the learning curve might require the technology to be in widespread use. If this knowledge is important to the decision maker, it would argue in favour of AWR even though the technology is not thought to be cost-effective on current evidence. However, a recent analysis based on over 40,000 patients administrative data from year 2006 to 2013 in Germany using hierarchical regression models showed the learning curve for fEVAR on in-hospital mortality and length of stay is actually negligible [25].

## Discussion

In this study we investigated the clinical and cost-effectiveness evaluation of fEVAR compared to open surgical repair as an example of a technology with limited evidence base and diffusion across a few specialized sites where the decision modelling is intended to identify the circumstances under which the new technology might represent a worthwhile investment and to inform future evidence generation projects.

This updated systematic review and meta-analysis found no RCTs that compared fEVAR and OSR. A recent NIHR funded project has updated a similar review up to March 2017 confirming no RCTs is available to date and the most recent comparative FEVAR vs OSR study was Raux et al. 2014 as per our results [59]. Seven observational studies compared these procedures, with varying methodological quality and substantial heterogeneity across all the outcomes.

Decision-makers responsible for setting prices of medical technologies or deciding re-imbursement policy need to make an assessment of whether the technology appears cost-effective on current evidence. In cases where the evidence base is highly uncertain and of poor methodological quality, decision makers may also need to assess what type of further research might be of value.

The usefulness of a decision model in these cases is not to make a definitive statement about cost-effectiveness, but rather to serve as a framework to organise the available data, identify the most important gaps in the evidence, and assess the plausible circumstances under which the technology might be cost-effective. In the fEVAR case, we were reliant on non-randomised comparative studies that are prone to bias and confounding. We assessed the risk of bias and focused our meta-analysis on four comparative studies that were judged to have performed adequate adjustment. These data were used in the model to estimate short-term outcomes. There was little data on late outcomes after fEVAR, particularly late AAA-deaths and complications. Long-term data from conventional endovascular devices clearly shows a greater rate of complications and re-interventions than after open repair, though it is difficult to know how generalizable these data are to fEVAR.

Using these data as base-case inputs, the model estimated that fEVAR is associated with greater survival and QALYs over a patient’s lifetime compared with OSR. However, the high acquisition cost of the device means that the cost of fEVAR considerably exceeds OSR. The EVAR-1 trial [22] showed that the frequency of re-interventions over the medium and long term is also likely to be greater under endovascular repair, though this is not a decisive driver of overall cost in the model. The overall treatment costs make fEVAR not cost-effective at conventional National Institute for Health and Care Excellence (NICE) thresholds, with an ICER exceeding £74,000/QALY. The cost-effectiveness threshold seeks to reflect the magnitude of health that other types of patients forgo when additional costs are incurred on a new technology rather than treatments from which they will benefit [24]. NICE’s threshold range of £20,000 to £30,000 has no empirical link to such opportunity costs, and recent research estimates the threshold to be lower at around £13,000 per QALY [60]. This would suggest that fEVAR is not cost-effective in the UK NHS based on current evidence. The model takes into account resource consumption estimates from the literature review of international studies, hence transferability of the model to other jurisdictions is possible, subject to availability of costs data from other countries. Criteria for decision-making rarely rely on a formal cost-effectiveness threshold outside UK [61], hence each decision maker would need to review and interpret these results in the context of the health systems in place in their jurisdiction. Sensitivity analyses showed that fEVAR would be close to being cost-effective at the upper range of NICE’s threshold if the OR of operative mortality were 0.19, as estimated by one of the studies [35]. fEVAR would also be cost-effective at this threshold if the price of the stent was about £7500 instead of £16,500. High-volume centres may already be able to negotiate discounts with suppliers, although these are commercially confidential.

Expected value of further information analysis showed that the population EVPI is not especially great, because this is a specialist procedure with a limited target population. This would increase if one was willing to accept that research is a global public good that would benefit patients worldwide [62]. Nevertheless, most of the decision uncertainty is around operative mortality. This might be effectively resolved by a short-term RCT, or possibly a well-conducted comparative observational study [59]. Decision makers may consider that a conditional coverage decision is warranted with assessments required to make this type of recommendation depending on local priorities and circumstances.

The model we have used in this paper is very simple, as we have tried to remain faithful to the evidence. There may be several other considerations which would warrant further evidence collection. For example, the model assumes the OR of operative mortality is independent of the base-line risk. In practice there may be sub-group effects. The literature review does not discriminate between male and female patients. There is some evidence from conventional EVAR that outcomes for females are different, given the different anatomical morphology. Over 90% of incident AAA patients between 55 and 84 years age are male, and outcomes and costs in women are insufficiently described in the literature [63]. There are some other important complications arising from cAAA repair, such as renal impairment or spinal cord injury that have not been taken into account in the model [8].

Nevertheless, the situation described in this study is typical of a medical-device procedure whose adoption and diffusion occur without strong clinical evidence supporting its marketing authorization, particularly in Europe. Based on current evidence, our model predicts that at conventional decision-making thresholds in the UK, fEVAR is not cost-effective and more research might plausibly reduce the decision uncertainty from the model. Staged approaches to evidence collection [64] or innovative and structured decision-making processes [16] linking the gathering of additional information with policies on approval for reimbursement around devices-related surgical procedures are recommended.

## Additional file

**Additional file 1.** **Appendix S1.** Search strategy. **Appendix S2.** List of included studies. **Appendix S3.** More on the Risk of bias for estimate of relative risk of operative mortality. **Table S1.** Inclusion and exclusion criteria. **Table S2.** Characteristics of included studies. **Table S3.** Risk of bias assessment. **Table S4.** Thirty-day mortality or peri-operative mortality in comparative studies. **Table S5.** Operative mortality and late mortality after fenestrated EVAR. **Figure S1.** Odds ratios of operative mortality fEVAR vs OSR, all comparative studies. **Figure S2.** Predicted survival curves from the base-case model. **Figure S3.** Predicted costs (£) per patient over time (undiscounted). **Figure S4.** Early re-interventions (in-hospital or 30 day), unadjusted odds ratio.
