# Supplementary material for: Decision uncertainty and value of further research: a case-study in fenestrated endovascular aneurysm repair for complex abdominal aortic aneurysms
Source: Cost Eff Resour Alloc. 2018 Apr 16;16:15. doi: 10.1186/s12962-018-0098-7 (PMC5902886; doi:10.1186/s12962-018-0098-7)
Supplement: Supplementary file 1 — Additional file 1. Appendix S1. Search strategy. Appendix S2. List of included studies. Appendix S3. More on the Risk of bias for estimate of relative risk of operative mortality. Table S1. Inclusion and exclusion criteria. Table S2. Characteristics of included studies. Table S3. Risk of bias assessment. Table S4. Thirty-day mortality or peri-operative mortality in comparative studies. Table S5. Operative mortality and late mortality after fenestrated EVAR. Figure S1. Odds ratios of operative mortality fEVAR vs OSR, all comparative studies. Figure S2. Predicted survival curves from the base-case model. Figure S3. Predicted costs (£) per patient over time (undiscounted). Figure S4. Early re-interventions (in-hospital or 30 day), unadjusted odds ratio. [file 12962_2018_98_MOESM1_ESM.docx]

**Additional material**

***Appendix S1: Search strategies***

Ovid MEDLINE(R) In-Process & Other N-Indexed Citations and Ovid MEDLINE(R) 1946 to Present 5 December 2014

**Search for Reviews**

1 aortic aneurysm/

2 Aortic aneurysm, abdominal/

3 1 or 2

4 (juxta-renal or juxtarenal or pararenal or para-renal or suprarenal or supra-renal or short-neck$ or shortneck$).ti,ab,ot,hw.

5 3 and 4

6 ((juxta-renal or juxtarenal) adj5 aneur?sm$).ti,ab,ot,hw.

7 ((pararenal or para-renal) adj5 aneur?sm$).ti,ab,ot,hw.

8 ((suprarenal or supra-renal) adj5 aneur?sm$).ti,ab,ot,hw.

9 ((short-neck$ or shortneck$) adj5 aneur?sm$).ti,ab,ot,hw.

10 (visceral aortic segment$ adj5 aneur?sm$).ti,ab,ot,hw.

11 6 or 7 or 8 or 9 or 10

12 (JRAAA or JRAAAs or PAAA or PAAAs or JPAA or JPAAs or SRA or SRAs or SRAA or SRAAs).ti,ab.

13 aneur?sm$.ti,ab.

14 12 and 13

15 5 or 11 or 14

16 (systematic adj review).ti,ab.

17 review.ti,ab.

18 (meta adj analysis).ti,ab.

19 meta-analysis.ti,ab.

20 guideline$1.ti,ab.

21 (review$3 or overview).ti,ab.

22 16 or 17 or 18 or 19 or 20 or 21

23 16 or 17 or 18 or 19 or 20 or 21

24 15 and 23

25 limit 24 to humans

26 limit 25 to english language

Ovid MEDLINE(R) In-Process & Other Nn-Indexed Citations and Ovid MEDLINE(R) 1946 to Present

14 January 2015

**Updated search for primary study**

1 aortic aneurysm/

2 Aortic aneurysm, abdominal/

3 Aortic aneurysm, thoracic/

4 1 or 2 or 3

5 (juxta-renal or juxtarenal or thoraco-abdominal or thoracoabdominal or thoracic abdominal or pararenal or para-renal or suprarenal or supra-renal or short-neck$ or shortneck$).ti,ab,ot,hw.

6 4 and 5

7 ((juxta-renal or juxtarenal) adj5 aneur?sm$).ti,ab,ot,hw.

8 ((thoraco-abdominal or thoracoabdominal or thoracic abdominal) adj5 aneur?sm$).ti,ab,ot,hw.

9 ((pararenal or para-renal) adj5 aneur?sm$).ti,ab,ot,hw.

10 ((suprarenal or supra-renal) adj5 aneur?sm$).ti,ab,ot,hw.

11 ((short-neck$ or shortneck$) adj5 aneur?sm$).ti,ab,ot,hw.

12 (visceral aortic segment$ adj5 aneur?sm$).ti,ab,ot,hw.

13 7 or 8 or 9 or 10 or 11 or 12

14 (JRAAA or JRAAAs or PAAA or PAAAs or TAAA or TAAAs or JPAA or JPAAs or SRA or SRAs or SRAA or SRAAs).ti,ab.

15 aneur?sm$.ti,ab.

16 14 and 15

17 6 or 13 or 16

18 letter.pt.

19 editorial.pt.

20 historical article.pt.

21 18 or 19 or 20

22 17 Not 21

23 limit 22 to humans

24 limit 23 to english language

25 limit 24 to yr="2013 -Current"

***Appendix S2: List of included studies***

1. Verhoeven EL, Vourliotakis G, Bos WT, Tielliu IF, Zeebregts CJ, Prins TR, Bracale UM, van den Dungen JJ. Fenestrated stent grafting for short-necked and juxtarenal abdominal aortic aneurysm: an 8-year single-centre experience. Eur J VascEndovasc Surg. 2010 May;39(5):529-36.

2. British Society for Endovascular Early results of fenestrated endovascular repair of juxtarenal aortic aneurysms in the United Kingdom. Therapy and the Global Collaborators on Advanced Stent-Graft Techniques for Aneurysm Repair (GLOBALSTAR) Registry. Circulation. 2012 Jun 5;125(22):2707-15.

3. Tambyraja AL, Fishwick NG, Bown MJ, Nasim A, McCarthy MJ, Sayers RD. Fenestrated aortic endografts for juxtarenal aortic aneurysm: medium term outcomes. Eur J VascEndovasc Surg. 2011 Jul;42(1):54-8.

4. Donas KP, Eisenack M, Panuccio G, Austermann M, Osada N, Torsello G. The role of open and endovascular treatment with fenestrated and chimney endografts for patients with juxtarenal aortic aneurysms.JVasc Surg. 2012 Aug;56(2):285-90.

5. Dijkstra ML, Tielliu IF, Meerwaldt R, Pierie M, van Brussel J, Schurink GW, LardeNye JW, Zeebregts CJ. Dutch experience with the fenestrated Anaconda endograft for short-neck infrarenal and juxtarenal abdominal aortic aneurysm repair. J Vasc Surg. 2014 Aug;60(2):301-7.

6. Vemuri C, Oderich GS, Lee JT, Farber MA, Fajardo A, Woo EY, Cayne N, Sanchez LA. Postapproval outcomes of juxtarenal aortic aneurysms treated with the Zenith fenestrated endovascular graft. J Vasc Surg. 2014 Aug;60(2):295-300.

7. Canavati R, Millen A, Brennan J, Fisher RK, McWilliams RG, Naik JB, Vallabhaneni SR. Comparison of fenestrated endovascular and open repair of abdominal aortic aneurysms Nt suitable for standard endovascular repair. J Vasc Surg. 2013 Feb;57(2):362-7.

8. Amiot S, Haulon S, Becquemin JP, Magnan PE, Lermusiaux P, Goueffic Y, Jean-Baptiste E, Cochennec F, Favre JP; Association Universitaire de RechercheenChirurgieVasculaire. Fenestrated endovascular grafting: the French multicenter experience.Eur J VascEndovasc Surg. 2010 May;39(5):537-44.

9. Greenberg RK, Sternbergh WC 3rd, Makaroun M, Ohki T, Chuter T, Bharadwaj P, Saunders A; Fenestrated Investigators. Intermediate results of a United States multicenter trial of fenestrated endograft repair for juxtarenal abdominal aortic aneurysms. J Vasc Surg. 2009 Oct;50(4):730-737.e1.

10. Ziegler P, Perdikides TP, AvgeriNs ED, Umscheid T, Stelter WJ. Fenestrated and branched grafts for para-anastomotic aortic aneurysm repair. J EndovascTher. 2007 Aug;14(4):513-9.

11. Semmens JB, Lawrence-Brown MM, Hartley DE, Allen YB, Green R, Nadkarni S. Outcomes of fenestrated endografts in the treatment of abdominal aortic aneurysm in Western Australia (1997-2004). J EndovascTher. 2006 Jun;13(3):320-9.

12. O'Neill S, Greenberg RK, Haddad F, Resch T, Sereika J, Katz E. A prospective analysis of fenestrated endovascular grafting: intermediate-term outcomes. Eur J VascEndovasc Surg. 2006 Aug;32(2):115-23.

13. Kristmundsson T, Sonesson B, Dias N, Tornqvist P, Malina M, Resch T. Outcomes of fenestrated endovascular repair of juxtarenal aortic aneurysm. J Vasc Surg. 2014 Jan;59(1):115-20

14. Moore Randy D, Macierewicz JA, Cina CS, Abuznadah W, Nutley M, Motamedi M. Fenestrated Endovascular vs Open Repair for Juxtarenal Aortic Aneurysmal Disease. Vascular Nvember 2006;14(Suppl 1):S122-23.

15. Kristmundsson T, Sonesson B, Malina M, Björses K, Dias N, Resch T. Fenestrated endovascular repair for juxtarenal aortic pathology. J VascSurg 2009;49:568-75.

16. Lee JT, Lee GK, Chandra V, Dalman RL. Comparison of fenestrated endografts and the snorkel/chimney technique. J Vasc Surg. 2014 Oct;60(4):849-56;

17. BanN H, Cochennec F, Marzelle J, Becquemin JP. Comparison of fenestrated endovascular aneurysm repair and chimney graft techniques for pararenal aortic aneurysm. J Vasc Surg. 2014 Jul;60(1):31-9

18. Rolls AE, Jenkins M, Bicknell CD, Riga CV, Cheshire NJ, Burfitt N, Hamady M. Experience with a novel custom-made fenestrated stent graft in the repair of juxtarenal and type IV thoracoabdominal aneurysms. J Vasc Surg. 2014 Mar;59(3):615-22.

19. Quiñones-Baldrich WJ, Holden A, Mertens R, Thompson MM, Sawchuk AP, Becquemin JP, Eagleton M, Clair DG. Prospective, multicenter experience with the Ventana Fenestrated System for juxtarenal and pararenal aortic aneurysm endovascular repair. J Vasc Surg. 2013 Jul;58(1):1-9.

20. Tsilimparis N, Perez S, Dayama A, Ricotta JJ 2nd Endovascular repair with fenestrated-branched stent grafts improves 30-day outcomes for complex aortic aneurysms compared with open repair. Ann Vasc Surg. 2013 Apr;27(3):267-73

21. Raux M, Patel VI, Cochennec F, Mukhopadhyay S, Desgranges P, Cambria RP, Becquemin JP, LaMuraglia GM. A propensity-matched comparison of outcomes for fenestrated endovascular aneurysm repair and open surgical repair of complex abdominal aortic aneurysms. J Vasc Surg. 2014 Oct;60(4):858-63.

22. Chisci E, Kristmundsson T, de Donato G, Resch T, Setacci F, Sonesson B, Setacci C, MalinaM.The AAA with a challenging neck: outcome of open versus endovascular repair with standard and fenestrated stent-grafts.JEndovascTher. 2009 Apr;16(2):137-46

*Appendix S3. More on the Risk of bias for estimate of relative risk of operative mortality*

Five studies reported statistically significant differences between the unmatched cohorts in one or more baseline variables, for example, in age [31,33], gender [34,35], or aneurysm size [30,33]. Hence the crude effect size may be biased. Two studies reported both adjusted and unadjusted odds ratios [30,35]. Tsilimparis reported that the crude odds ratio showed a greater benefit for fEVAR than the adjusted odds ratio [35], whereas in the Canavati study [30], the adjusted odds ratio showed a greater effect than the crude effect size. Without further information, we cannot determine the direction of the bias in the crude effect size in the other studies.

Two studies used a published risk score [30,36], the Vascular Physiological and Operative Severity Score for enumeration of Mortality and Morbidity (V-POSSUM 37) to estimate the expected number of operative deaths under OSR in the fEVAR group, and then calculate the adjusted odds ratio using the observed proportion of deaths in the fEVAR group and the expected proportion using the risk score, if these patients had received OSR. However, using a risk score prediction as if it were known data overestimates the precision, and so we considered this methodology presents a moderate risk of bias in this population.

Raux and colleagues used propensity score matching to try to select a subset of patients who would have been suitable for either operation [34]. The authors identified controls who matched fEVAR patients in a 4:1 ratio on gender, age, chronic obstructive pulmonary disease (COPD), coronary artery disease (CAD), congestive heart failure (CHF), history of coronary intervention, diabetes, and actual or anticipated clamp location, among other variables. Propensity score matching is often considered an acceptable method of handling selection bias. However, the study design has been criticised. The authors reported that the fEVAR patients recruited to this study were all classified by their surgeon as unfit for open surgery. If there does not exist even in principle any subgroup of the sample who would have been fit for either procedure, then any matching algorithm will be invalid. If this is the case, then the poor results after fEVAR can still be attributed to the more complex case-mix in this group. On the other hand, all the fEVAR in this study were carried out at a centre in the United States while all the OSR were carried out at a centre in France. As a surgeon’s assessment of a patient’s fitness for open surgery is in part subjective, the threshold for fitness for open surgery may vary between surgeons and centres. This variability could allow the authors to identify a suitably matched subgroup. One still cannot however rule out the possibility that the teams in one centre were simply more skilled or experienced that the other.

*Table S1 Inclusion and exclusion criteria for systematic review*

| Inclusion criteria | Exclusion criteria |
| --- | --- |
| 1) Study design: Systematic reviews and meta-analyses (as a way to access primary studies) or RCTs, Non-RCTs, observational studies (including comparative and non-comparative designs)  2) Intervention: fEVAR*  3) Comparison: OSR (if non-comparative)  4) Study population: Adult patients (≥18 years) who were eligible for fEVAR with cAAAs, such that standard EVAR was unsuitable  5) Outcomes: i) mortality (perioperative/up to 30-days post operation and overall) AND/OR ii) type and frequency of complications and re-intervention (up to 30-day post operation and overall) AND/OR iii) HRQoL or utility measures AND/OR iv) in-hospital resource consumption (e.g. length of stay, consumables, time in intensive care unit, operating time, blood loss) AND/ORcosts. | 1) Languages other than English  2) Sample size below 10 patients  3) Studies not presenting any of the outcomes of interest  4) Duplicated studies or older cohorts updated by more recent reports (the latest report is included)  5) Studies focused on fEVAR performed as rescue therapy after previous open or standard EVAR  6) Studies focusing on symptomatic patients/emergency procedure  7) Eligibility criteria not appropriate to the study question (e.g. indication for branched EVAR)  8) Inadequate adjustment for confounding factors in the clinical effectiveness evaluation |

HRQoL = Health related quality of life; RCT = randomized controlled trial

* as described by Greenberg RK, Lytle B. Endovascular repair of thoracoabdominal aneurysms. Circulation 2008; 117: 2288-96

*Table S2 Characteristics of included studies*

|  | First Author | Year | Country | Median follow-up (months) | Sample size | Mean age (years) | Male:Female (count) | Anatomical location of aneurysms | Device |
| --- | --- | --- | --- | --- | --- | --- | --- | --- | --- |
| Case series | | | | | | | | | |
| 1 | Semmens | 2006 | Australia (Multicentre) | 16.8* | 58 | 75.5 | 51:7 | AAA | Zenith fenestrated device (Cook Ltd, Brisbane, Australia) |
| 2 | O’Neill | 2006 | US (Cleveland) | 19* | 119 | 75 | 98:21 | Juxtarenal AAA | Zenith fenestrated device (Cook Inc, Bloomington, Ind) |
| 3 | Ziegler | 2007 | Germany (Frankfurt) | 14 | 63 | 70.5 | 57:6 | Para-anastomotic, thoracoabdominal, thoracic AAA | Zenith fenestrated device (Cook Ltd, Brisbane, Australia) |
| 4 | Greenberg | 2009 | US (Multicentre) | 24 | 30 | 75 | 24:6 | Juxtarenal AAA | Zenith fenestrated device (Cook Inc, Bloomington, Ind) |
| 5 | Verhoeven | 2010 | Netherlands (Groningen) | 24 | 100 | 72.6 | 87:13 | Short-necked or juxtarenal AAA | Zenith fenestrated device (Cook Inc, Bloomington, Ind) |
| 6 | Amiot | 2010 | France (Multicentre) | 15 | 134 | 73¶ | 129:5 | Juxtarenal, suprarenal or type IV thoraco-AAA | Zenith fenestrated device (Cook Inc, Bloomington, Ind) |
| 7 | Tambyraja | 2011 | UK (Leicester) | 20 | 29 | 74¶ | 27:2 | Juxtarenal AAA | Zenith fenestrated device (Cook Inc, Bloomington, Ind) |
| 8 | Quiñones-Baldrich | 2013 | Chile France New Zealand UK US | 15.6* | 31 | 73 | 28:3 | Juxtarenal and pararenal AAA | Ventana fenestrated stent graft (EndologixInc, Irvine***, Calif)*** |
| 9 | Kristmundsson | 2013 | Sweden (Malmö) | 25 | 88 | 70¶ | 69:19 | Juxtarenal AAA | Zenith fenestrated device (Cook ApS, Bjaeverskov, Europe) |
| 10 | Dijkstra | 2014 | Netherlands (Multicentre) | 11 | 25 | 73 | 22:3 | Juxtarenal or short-neck AAA | Anaconda Endograft (Vascutek Ltd, Renfrewshire, Scotland) |
| 11 | Vemuri | 2014 | US (Multicentre) | 1.8* | 57 | 73.3 | 46:11 | Juxtarenal AAA | Zenith fenestrated device (Cook Inc, Bloomington, Ind) |
| 12 | Kristmundsson | 2014 | Sweden (Malmö) | 67 | 54 | 72¶ | 46:8 | Juxtarenal AAA | Zenith fenestrated device (Cook Inc, Bloomington, Ind) |
| 13 | Lee | 2014 | US (Stanford) | Min 6 | 15 | 77.4 | 10:5 | Short-neck or juxtarenal AAA | Zenith fenestrated device (Cook Inc, Bloomington, Ind) |
| 14 | Banno | 2014 | France (Creteil) | 14 | 80 | 73.9 | 72:8 | Pararenal AAA | Zenith fenestrated device (Cook Inc, Bloomington, Ind)  Ventana fenestrated stent graft (EndologixInc, Irvine, Calif) Anaconda Endograft (Vascutek Ltd, Renfrewshire,***Scotland)*** |
| 15 | Rolls | 2014 | UK (London) | 12 | 13 | 75¶ | 8:5 | Juxtarenal, pararenal, type IV thoraco AAA, two or more unfavorable anatomical features | Anaconda Endograft (Vascutek Ltd, Renfrewshire, Scotland) |
| Comparative studies | | | | | | | | | |
| 16 | Moore | 2006 | Canada | Min 1 | 16 | 77 | 15:1 | Juxtarenal AAA | Zenith fenestrated device (Cook Inc, Bloomington, Ind) |
| 17 | Chisci | 2009 | Italy Sweden | 13.9* | 52 | 71.5 | 42:10 | Superior mesenteric artery, renal arteries, all visceral vessels with asymptomatic AAA with challenging proximal neck | Zenith fenestrated device (Cook Ltd, Brisbane, Australia) |
| 18 | Donas | 2012 | Germany (Munster) | 13.2* | 29 | 73.7 | 29:0 | Juxtarenal AAA | Zenith fenestrated device (Cook Inc, Bloomington, Ind) |
| 19 | GLOBALSTAR | 2012 | UK (Multicentre) | 6 | 318 | 74 | 273:45 | Juxtarenal and pararenal AAA | Zenith fenestrated device (Cook Inc, Bloomington, Ind) |
| 20 | Canavati | 2013 | UK (Liverpool) | NR | 53 | 72¶ | 47:6 | Juxtarenal AAA | Zenith fenestrated device (Cook Inc, Bloomington, Ind) |
| 21 | Tsilimparis | 2013 | US (Multicentre) | Min 1 | OSR = 1091 fEVAR = 264 | OSR = 71 fEVAR = 74 | OSR = 780:311 fEVAR= 217:47 | cAAA | Customized and surgeon-modified stent grafts |
| 22 | Raux | 2014 | US  France | Min 1 | ‡OSR = 147 fEVAR = 42 | OSR = 73  fEVAR = 73 | OSR = 120:27  fEVAR = 37:5 | cAAA | Zenith fenestrated device (Cook Ltd, Brisbane, Australia) |

* Mean follow-up

§ Median age

‡ Propensity-matched dataset

***Table S3. Risk of bias assessment***

|  | 1 | 2 | 3 | 4 | 5 | 6 | 7 | 8 | 9 | 10 | 11 | 12 | 13 | 14 | 15 | 16 | 17 | 18 | 19 | 20 | 21 |
| --- | --- | --- | --- | --- | --- | --- | --- | --- | --- | --- | --- | --- | --- | --- | --- | --- | --- | --- | --- | --- | --- |
| FEVAR Case series | | | | | | | | | | | | | | | | | | | | | |
| Semmens 2006 | Y | N | Y | ? | ? | ? | Y | N | Y | Y | NA | NA | Y | Y | N | Y | Y | N | NA | NA | NA |
| O'Neill 2006 | Y | Y | N | ? | ? | ? | Y | N | Y | Y | NA | NA | Y | N | N | Y | Y | Y | NA | NA | NA |
| Ziegler 2007 | Y | Y | N | ? | Y | ? | Y | N | Y | Y | NA | NA | Y | N | N | N | Y | Y | NA | NA | NA |
| Greenberg 2009 | Y | Y | Y | Y | ? | ? | Y | N | Y | Y | NA | NA | Y | N | N | Y | Y | Y | NA | NA | NA |
| Verhoeven 2010 | Y | Y | N | ? | Y | ? | Y | N | Y | Y | NA | NA | Y | Y | N | Y | Y | N | NA | NA | NA |
| Amiot 2010 | Y | Y | Y | ? | Y | ? | Y | N | Y | Y | NA | NA | Y | Y | N | Y | Y | Y | NA | NA | NA |
| Tambyraja 2011 | Y | Y | N | ? | Y | ? | Y | N | Y | Y | NA | NA | Y | Y | N | Y | Y | Y | NA | NA | NA |
| Quiñones-Baldrich 2013 | Y | Y | Y | Y | ? | ? | Y | N | Y | Y | NA | NA | Y | Y | N | Y | Y | Y | NA | NA | NA |
| Kristmundsson 2013 | Y | Y | N | ? | N | ? | Y | N | Y | Y | NA | NA | N | N | N | Y | Y | Y | NA | NA | NA |
| Dijkstra 2014 | Y | Y | Y | ? | Y | ? | Y | N | Y | Y | NA | NA | Y | N | N | Y | Y | Y | NA | NA | NA |
| Vemuri 2014 | Y | Y | Y | ? | Y | ? | Y | N | Y | Y | NA | NA | Y | Y | N | Y | Y | Y | NA | NA | NA |
| Kristmundsson2014 | Y | Y | N | ? | Y | ? | Y | N | Y | Y | NA | NA | Y | Y | N | Y | Y | Y | NA | NA | NA |
| Lee 2014 | Y | Y | N | ? | Y | ? | Y | N | Y | Y | NA | NA | Y | N | N | Y | Y | Y | NA | NA | NA |
| Banno 2014 | Y | Y | N | ? | Y | ? | Y | N | Y | Y | NA | NA | Y | Y | N | Y | Y | Y | NA | NA | NA |
| Rolls 2014 | Y | Y | N | ? | ? | ? | Y | N | Y | Y | NA | NA | Y | N | N | Y | Y | Y | NA | NA | NA |
| Comparative studies FEVAR vs OSR | | | | | | | | | | | | | | | | | | | | | |
| Moore 2006 | Y | N | ? | ? | ? | ? | N | N | Y | Y | NA | N | N | N | N | Y | Y | N | N | N | ? |
| Chisci 2009 | Y | Y | Y | Y | Y | ? | Y | N | Y | Y | NA | N | Y | Y | N | Y | Y | Y | N | N | ? |
| Donas 2012 | Y | Y | N | ? | ? | N | Y | N | Y | Y | NA | N | Y | N | N | Y | Y | Y | N | N | ? |
| GLOBALSTAR 2012 | Y | Y | Y | ? | Y | ? | Y | N | Y | Y | NA | Y | Y | N | N | Y | Y | Y | Y | Y | ? |
| Canavati 2013 | Y | Y | N | ? | Y | Y | Y | N | Y | Y | NA | Y | N | Y | N | Y | Y | Y | Y | Y | ? |
| Tsilimparis2013 | Y | Y | Y | Y | ? | ? | Y | N | Y | Y | NA | Y | N | N | Y | Y | Y | N | Y | Y | N |
| Raux 2014 | Y | Y | Y | N | ? | N | Y | N | Y | Y | NA | Y | N | N | Y | Y | Y | Y | Y | Y | Y |

1. Is the hypothesis/aim/objective of the study clearly stated in the abstract, introduction or methods section?

2. Are the characteristics of the participants included in the study described?

3. Were the cases collected in more than one centre?

4. Are the eligibility criteria (inclusion and exclusion criteria) to entry the study explicit and appropriate?

5. Were participants recruited consecutively?

6. Did participants enter the study at a similar point in the disease?

7. Was the intervention clearly described in the study?

8. Were additional interventions (co-interventions) clearly reported in the study?

9. Are the outcome measures clearly defined in the introduction or methods section? (30-day mortality)

10. Were relevant outcomes appropriately measured with objective and/or subjective methods? (30-day mortality)

11. Were outcomes measured before and after intervention? (30-day mortality)

12. Were the statistical tests used to assess the relevant outcomes appropriate?

13. Was the length of follow-up reported?

14. Was the loss to follow-up reported?

15. Does the study provide estimates of the random variability in the data analysis of relevant outcomes?

16. Are adverse events reported? (30-day follow-up)

17. Are the conclusions of the study supported by results?

18. Are both competing interest and source of support for the study reported?

*19. Have the authors taken account of potential confounding factors in the design or analyses?*

*20. Are results adjusted for confounding?*

*21. Has adjustment made a big difference?*

*Table S4 Thirty-day mortality or peri-operative mortality in comparative studies*

|  | Unadjusted outcome | Unadjusted outcome | Unadjusted odds ratio | Adjusted outcome | Adjusted odds ratio | Method of adjustment | Endpoint in study |
| --- | --- | --- | --- | --- | --- | --- | --- |
| Study | fEVAR | OSR |  | OSR |  |  |  |
| Moore 2006 | 1/16 | 2/29 | 0.90 95% CI 0.08-10.77 | n.a. | n.a. | None undertaken | 30 day |
| Chisci 2009 | 3/52 | 2/61 | 1.81 95% CI 0.29-11.25 | n.a. | n.a. | None undertaken | 30 day |
| Donas 2012 | 0/29 | 2/31 | 0.20 95% CI 0.01-4.35 | n.a. | n.a. | None undertaken | 30 day |
| GLOBALSTAR 2012 | 13/318 | n.a. | n.a. | 27/246 | OR 0.39 95% CI 0.20 0.77 | Predicted deaths (V-POSSUM) in fEVAR group if they had undergone open repair | Perioperative |
| Canavati 2013 | 2/53 | 5/54 | 0.38 95% CI 0.07-2.08 | 7/53 | OR 0.26 95% CI 0.05-1.34 | Predicted deaths (V-POSSUM) in fEVAR group if they had undergone open repair | Perioperative |
| Tsilimparis 2013 | 2/264 | 59/1091 | 0.13 95% 0.03-0.55 | n.a. | OR 0.192 95% CI 0.045-0.833 | Adjusted in multivariate analysis for age, body mass index, estimated glomerular filtration rate, perioperative international normalized ratio | 30 day |
| Raux 2014 | 4/42 | 3/147 | OR 5.1; 95% CI 1.1-24 | n.a. | OR 5.1; 95% CI 1.1-24 | All results were shown in the paper for patients matched using propensity score. The authors report that the multivariate analysis model gave the same results as the univariate analysis model. | 30 day |

*Table S5. Operative mortality and late mortality after fenestrated EVAR*

| ID | Operative deaths | N | Operativemortality rate | Late AAA deaths | Follow up person-years | Late AAA mortality rate (deaths/py) |
| --- | --- | --- | --- | --- | --- | --- |
| Verhoeven 2010 | 1 | 100 | 0.010 | 0 | 325.3 | 0 |
| GLOBALSTAR 2012 | 13 | 318 | 0.041 | 0 | 250.5 | 0 |
| Tambyraja 2011 | 0 | 29 | 0.000 | 0 | 79.4 | 0 |
| Donas 2012 | 0 | 29 | 0.000 | 0 | 31.9 | 0 |
| Dijkstra 2014 | 0 | 25 | 0.000 | 0 | 37.6 | 0 |
| Vemuri 2014 | 1 | 57 | 0.018 |  |  | n.r. |
| Canavati 2013 | 2 | 53 | 0.038 |  |  | n.r. |
| Amiot 2010 | 3 | 134 | 0.022 |  |  | n.r. |
| Greenberg 2009 | 0 | 30 | 0.000 | 0 | 98.6 | 0 |
| Ziegler 2007 | 2 | 63 | 0.032 | 2 | 118.8 | 0.017 |
| Semmens 2006 | 2 | 58 | 0.034 |  |  | n.r. |
| O’Neill 2006 | 1 | 119 | 0.008 | 0 | 186.8 | 0 |
| Kristmundsson 2009 | 2 | 54 | 0.037 | 3 | 178.0 | 0.017 |
| Moore 2006 | 1 | 16 | 0.063 |  |  | n.r. |
| Kristmundsson 2013 | 1 | 88 | 0.011 |  |  | n.r. |
| Lee 2014 | 0 | 15 | 0.000 | 0 | 12.3 | 0 |
| Banno 2014 | 5 | 80 | 0.063 | 1 | 138.0 | 0.007 |
| Rolls 2014 | 0 | 13 | 0.000 |  |  | n.r. |
| Quiñones-Baldrich 2013 | 0 | 31 | 0.000 | 0 | 40.3 | 0 |
| Tsilimparis 2013 | 2 | 264 | 0.008 |  |  | n.r. |
| Raux 2014 | 4 | 42 | 0.095 |  |  | n.r. |
| Chisci 2009 | 3 | 52 | 0.058 | 0 | 60.2 | 0 |
| Total | 43 | 1670 | 0.026 | 6 | 1557.8 | 0.004 |

AAA = abdominal aortic aneurysms; n.r. = not reported

*Figure S1. Odds ratios of operative mortality fEVAR vs OSR, all comparative studies*

*Figure S2. Predicted survival curves from the base-case model*

***Figure S3. Predicted costs (£) per patient over time (undiscounted)***

| Year | Cost OSR, £ | Cost fEVAR, £ |
| --- | --- | --- |
| 0-6m | 12,816 | 26,453 |
| >6m-4yr | 202 | 1,273 |
| >4yr-8yr | 498 | 797 |
| >8yr | 258 | 404 |

Notes: OSR: Open surgical repair; fEVAR: fenestrated endovascular repair.

***Figure S4. Early re-interventions (in-hospital or 30 day), unadjusted odds ratio***
